# Long-Term Exposure–Recovery to 20 nm Polystyrene Nanoplastic Particles Is Associated with Residual Nuclear Stress in a Marine Fish Cell Line

**DOI:** 10.3390/toxics14070628

**Published:** 2026-07-20

**Authors:** Lulu Yan, Jiaqi Su, Changbo Zhu

**Affiliations:** College of Animal Science, Inner Mongolia Agricultural University, Hohhot 010018, China; luluyan1103@gmail.com (L.Y.); sujiaqi2005@126.com (J.S.)

**Keywords:** nanoplastics, *Lateolabrax maculatus*, nucleus, genotoxicity, nuclear envelope

## Abstract

Nanoplastic particles are increasingly detected in aquatic environments, yet whether cellular stress responses persist after exposure cessation remains unclear. Here, *Lateolabrax maculatus* rostral (LMR) cells from spotted sea bass were continuously exposed for up to 30 passages (105 days) to nominal 20 nm carboxylated fluorescent polystyrene particles (20 μg/mL), followed by 10 particle-free passages (recovery). Long-term exposure was associated with reduced proliferation and pronounced changes in cell surface morphology and ultrastructure. Although NP-associated fluorescence became undetectable during recovery, several nuclear-associated alterations persisted throughout the 10-passage recovery period, including TEM-observed nuclear-envelope alterations, filamentous actin (F-actin) reorganization, and sustained elevation of phosphorylated H2AX (γ-H2AX) foci. Several nuclear pore complex (NPC) genes were downregulated during exposure and rebounded after particle removal, whereas the nuclear-to-cytoplasmic distribution of proliferating cell nuclear antigen (PCNA) remained shifted. Together, these findings indicate that prolonged exposure to this nanoscale polystyrene particle formulation was associated with nuclear stress responses that did not fully resolve within the 10-passage recovery window in fish cells. Because bulk-polymer and chemical-extract (leachate) controls were not included, nanoscale-specific effects cannot be distinguished from contributions of polymer chemistry, surface functionalization, fluorescent dye, or other formulation-related effects.

## 1. Introduction

Although plastics have been used at scale for only a little more than a century, their environmental and biological impacts have far exceeded early expectations. Once in aquatic environments, plastic debris undergoes physical, chemical, and biological fragmentation to form microplastics (MPs, particles < 5 mm) and nanoplastics (NPs, particles < 100 nm) [1,2]. A 2025 basin-scale survey detected polyethylene terephthalate (PET), polystyrene (PS), and polyvinyl chloride (PVC) particles in the <1 μm size fraction throughout the North Atlantic water column and estimated approximately 27 million tonnes in the mixed layer of the temperate-to-subtropical North Atlantic, highlighting the potentially underestimated mass of nanoscale plastic pollution [3]. The marine environment is a critical habitat for numerous fish species, which can ingest and accumulate these particles and experience repeated or prolonged exposure [4,5,6]. NPs are of particular concern because their small size facilitates interactions with biological barriers, cell membranes, and intracellular structures [7]. Among common polymers, PS, polyethylene (PE), polypropylene (PP), and PVC are frequently reported in environmental plastic debris, and PS is widely used as an experimental model for nanoplastic toxicity [8].

In recent years, substantial progress has been made in understanding NP toxicity in fish. Studies have reported impaired growth and development, altered behavior, compromised immune function, and reproductive dysfunction after NP exposure [1,9,10,11]. NP exposure has also been associated with oxidative stress, inflammation, genotoxicity, and altered gene expression and signaling pathways in fish [1,12]. In vitro studies using fish and other cell-line models have provided complementary mechanistic evidence, including changes in cytoskeletal organization, cell morphology, membrane integrity, and subcellular structure [13,14]. NPs may also interact with cell-surface receptors and membrane proteins, potentially affecting cellular signaling and communication [13,15,16]. Nevertheless, most in vitro studies still emphasize acute or medium-term exposure windows. Less is known about how cellular responses evolve during repeated subculture under continuous NP exposure or whether stress-associated phenotypes are fully reversible after particle exposure ceases.

To address these questions, we used a spotted sea bass (*Lateolabrax maculatus*) rostral (LMR) cell line in a two-part experimental design. Experiment 1 was a short-term concentration- and time-dependent screen in 96-well plates, using MTT-based viability assessment and bright-field cytopathic-effect grading to identify a concentration suitable for prolonged exposure. Experiment 2 then exposed cells to 20 μg/mL NPs during 30 successive cell-subculture passages over approximately 105 days in T-25 flasks. After the 30th passage, NP addition was discontinued and cells were passaged for 10 additional particle-free passages to evaluate recovery. The main objectives were to determine whether prolonged NP exposure altered proliferation, morphology, ultrastructure, NP-associated fluorescence, DNA damage markers, oxidative-stress indices, and nuclear-associated molecular endpoints, and whether these changes resolved after exposure cessation.

## 2. Materials and Methods

### 2.1. Cell Line and Culture Conditions

We used the *L. maculatus* rostral (LMR) cell line, a laboratory-established fish cell line that supports stable proliferation during long-term passaging. The cell line had been passaged 40 times before the present experiment, and its species origin had been confirmed as *L. maculatus* by 18S rRNA gene sequencing during cell-line establishment [17]. Routine culture was performed at 28 °C. A serum concentration of 25% fetal bovine serum (FBS) was used for routine expansion of the LMR cell line, 20% FBS complete medium was used for long-term serial passaging in Experiment 2, and 5% FBS maintenance medium was used in the short-term 96-well toxicity assay in Experiment 1 to maintain basal cell survival while reducing serum-related masking of toxicity.

### 2.2. Nanoplastic Particles and Characterization

We purchased red carboxylated fluorescent polystyrene microspheres functionalized with carboxyl (COOH) groups as a suspension from HuicH (Shanghai, China). The suspension had a solids content of 1% (10 mg/mL) and consisted of deionized water with trace amounts of surfactant. The microspheres consisted of a red-dye-embedded polystyrene copolymer with a nominal diameter of 20 nm and a density of 1.05 g/cm^3^. The refractive index was 1.59 (at a wavelength of 589 nm, 25 °C). The excitation and emission wavelengths were 535 and 610 nm, respectively. The particles were negatively charged and had a stated shelf life of five years. Because of their small diameter, we examined NP morphology using scanning electron microscopy (SEM), and we measured the diameter of 500 randomly selected NPs using ImageJ [18]. In addition, we used a Zetasizer Nano ZS (Malvern, Worcestershire, UK) to measure the hydrodynamic diameter of the red carboxylated fluorescent polystyrene microspheres by dynamic light scattering (DLS) in both deionized water and complete culture medium. Each sample was measured three times.

To investigate the fluorescence stability of polystyrene nanoplastics (NPs), their photoluminescence (PL) spectra were measured using a Horiba FluoroMax-4 luminescence spectrometer (Horiba Scientific, Kyoto, Japan). The NPs (20 μg/mL in complete medium) were tested under two conditions: (1) complete light exclusion for 35 days and (2) intermittent light exposure over 35 days, simulating 10 rounds of cell passaging, with exposure occurring approximately every 3.5 days, each exposure lasting 3 min, and a total exposure time of 30 min. The PL spectra were recorded with an excitation wavelength of λex = 535 nm and an emission range of λem = 555–700 nm, using a slit width of 2 nm. The results were compared with the initial NP PL spectra recorded before the experiment. This fluorescence-stability test was designed as a study-specific validation of particle fluorescence under the light-exclusion and intermittent-handling conditions used during culture and recovery.

### 2.3. Experimental Design

The exposure design consisted of a short-term concentration screen followed by prolonged exposure and recovery assessment. The concentration range in Experiment 1 was selected to cover low to high nominal particle concentrations. The 20 μg/mL concentration used in Experiment 2 was selected empirically because it produced a gradual cytotoxic response in Experiment 1 while still allowing continued serial passaging. Accordingly, this concentration was used as a biologically informative exposure level for evaluating cumulative cellular responses and post-exposure recovery (Figure S1).

The NP exposure experiments in LMR cells were conducted as follows:

Experiment 1 was performed directly in 96-well cell culture plates to analyze concentration- and time-dependent cytotoxicity. LMR cells were seeded at 1 × 10^4^ cells/well. Before seeding, cells from independent T-25 culture flasks were enzymatically detached, thoroughly resuspended, and counted using a Countstar Mira BF automated cell analyzer (Countstar, Shanghai, China). Cell suspensions were adjusted to the target density before plating, mixed again immediately before dispensing, and seeded into inner wells only to minimize edge effects. Cells were then exposed to L-15 medium containing 5% FBS and 0 (control), 2, 20, 200, or 2000 μg/mL NPs. Cell morphology and viability were assessed on days 1, 3, 7, and 14. For each concentration–time point combination, five assay wells were analyzed; each well was seeded from a separately maintained T-25 culture flask, and these five flasks were treated as independent biological replicates for the short-term 96-well assay. The cell culture medium was changed every 3 days without subculturing the cells.

For Experiment 1, cytotoxicity was assessed using a six-level cytopathic-effect (CPE) grading approach adapted from our previous study [19]. The specific thresholds were predefined for the present experimental system by integrating the reduction in MTT-measured cell viability with bright-field morphological evidence of CPEs: L0, no cytotoxicity, with unchanged cell viability and no CPEs; L1, <20% reduction in cell viability with CPEs; L2, 20–40% reduction with CPEs; L3, 40–60% reduction with CPEs; L4, 60–90% reduction with CPEs; and L5, >90% reduction with CPEs.

Experiment 2 was designed to evaluate prolonged exposure during serial subculture and subsequent recovery after exposure cessation. We initiated three independent founder T-25 flask lineages for the NP-exposed group and three independent founder T-25 flask lineages for the time-matched control group. Each flask was seeded with 7 × 10^6^ cells. NP-exposed flasks contained complete medium (L-15 medium supplemented with 20% FBS) with 20 μg/mL NPs, whereas control flasks contained complete medium without NPs. In this context, one passage refers to one complete subculture cycle: cells were grown to 80–90% confluence, enzymatically detached, thoroughly mixed, counted using the Countstar Mira BF automated cell analyzer, and reseeded at approximately 7 × 10^6^ cells per new T-25 flask. For routine maintenance, one daughter flask from each founder lineage was retained for continued passaging. At sampling passages, additional daughter flasks were retained from the corresponding founder lineages and clearly tracked by lineage origin to provide assay material and reduce the risk of sample loss. After the 30th passage in the NP-exposed group, the NP-containing medium was removed, NP addition was stopped, and cells were cultured and passaged for 10 additional passages in particle-free complete medium as the recovery group. At each sampling passage requiring five assay flasks per group, the five flasks were derived from the three original founder lineages with a recorded 1:2:2 distribution (one flask from lineage 1, two flasks from lineage 2, and two flasks from lineage 3).

Throughout the experiment, we conducted sampling at predefined time points and measured cell doubling time, cell viability, NP-associated fluorescence, DNA double-strand break markers, F-actin morphology, cell-surface ultrastructure, subcellular ultrastructure, cell proliferation, and nuclear gene and protein endpoints. Unless otherwise specified, quantitative assays were based on independently handled flask lineages or on daughter flasks with a documented founder-lineage origin. When multiple daughter flasks originated from the same founder lineage at a sampling passage, their lineage origin was retained in the experimental record, and subsampling units such as wells, fields of view, or individual cells were not treated as independent biological replicates for statistical interpretation. The purchased NP suspension (10 mg/mL) was sterilized by filtration through a 0.22 μm membrane filter. Before dilution into culture medium, the stock suspension and working NP suspensions were thoroughly vortex-mixed. For the experimental group, filtered NPs were freshly added to cell culture flasks or 96-well plates after each passage or medium change. Because the suspension contained only trace surfactant, an equivalent volume of sterile water was added to the control group. To avoid fluorescence quenching, all cells were protected from light by wrapping the culture flasks or plates with aluminum foil. NP-exposed samples were designated as “NP + passage number or treatment duration,” and recovery samples were designated as “RCY + passage number or time after NP discontinuation”; for example, NP-20th refers to cells after 20 passages of NP exposure. Accordingly, Experiment 2 endpoints reported as n = 5 represent five subculture-derived assay flasks nested within three founder lineages, not five fully independent founder cultures.

### 2.4. Cell Toxicity Assessment

In Experiment 1, cell viability was assessed using the methylthiazolyldiphenyl-tetrazolium bromide (MTT) assay kit (Solarbio, Beijing, China) according to the manufacturer’s instructions and a previously published method [19]. Blank wells containing medium and MTT reagent but no cells were included. Absorbance was measured at 490 nm using a Spark microplate reader (Tecan, Männedorf, Switzerland), and viability was calculated as [(experimental well − blank well)/(negative control well − blank well)] × 100%. Because the 96-well assay used inner wells only and cells were counted and normalized before seeding, well-to-well differences in initial cell number were minimized. Cell morphology was observed using an inverted fluorescence microscope (Leica DMI8, Leica Microsystems, Wetzlar, Germany), and cytopathic effects (CPEs) were recorded.

For cells treated with 20 μg/mL NPs in T-25 flasks, cell doubling time was measured at passages 10, 20, and 30. Cells were detached, mixed thoroughly, counted using the Countstar Mira BF automated cell analyzer, adjusted to the target density, and seeded into inner wells of 96-well plates at 1 × 10^4^ cells/well. After 4 h, non-adherent cells were removed, and the remaining adherent cells were subjected to the MTT assay to obtain the initial optical density (OD; N0). After an additional 48 h of incubation, OD was measured again (Nt). Doubling time was calculated as 48 × lg2/(lgNt − lgN0), where N0 is the OD at the initial post-attachment time point and Nt is the OD after 48 h [20,21]. The n = 9 values shown for doubling-time analysis represent tracked subculture-derived assay samples originating from the three founder lineages.

### 2.5. Cellular Uptake and Immunofluorescence Analysis

To examine NP-associated fluorescence patterns during exposure and recovery, we collected cell samples from NP-1st, NP-10th, NP-30th, RCY-48h, RCY-5th, and RCY-10th. Cells were seeded onto round coverslips, fixed with 4% paraformaldehyde for 20 min, washed with phosphate-buffered saline (PBS), stained with 4′,6-diamidino-2-phenylindole (DAPI), and mounted with anti-fade reagent. Images were captured using a fluorescence microscope (Nikon Eclipse C1, Tokyo, Japan) and a confocal laser scanning microscope (Nikon Eclipse Ti) at specified excitation (EX) and emission (EM) wavelengths (DAPI, EX 330–380 nm, EM 420 nm; NPs, EX 535 nm, EM 610 nm). Five subculture-derived assay flasks nested within three founder lineages were measured (lineage distribution: one flask from lineage 1, two flasks from lineage 2, and two flasks from lineage 3), with 20 random fields of view selected for each assay flask. NP fluorescence area, NP fluorescence density, and cell count were quantified using ImageJ software (https://imagej.net/ accessed on 3 June 2025, version 1.53) to calculate average NP-associated fluorescence area and density per cell [18].

We seeded cell samples from the NP-30th, RCY-10th, and control groups onto round coverslips with a diameter of 20 mm. Cells were fixed with 4% paraformaldehyde and then washed with PBS. Permeabilization was achieved with 0.5% Triton X-100 for 20 min at room temperature, followed by three PBS washes. Cells were blocked with 3% bovine serum albumin for 30 min at room temperature and subsequently incubated overnight at 4 °C with Phospho-H2A histone family member X (γ-H2AX) rabbit polyclonal antibody or mouse anti-proliferating cell nuclear antigen (PCNA) polyclonal antibody (Servicebio, Wuhan, China). After three PBS washes, we incubated the cells with IF488-labeled goat anti-rabbit immunoglobulin G (IgG) (Servicebio) at room temperature for 50 min. After three PBS washes, cells were stained with DAPI for 10 min at room temperature in the dark and mounted with antifade mounting medium. Imaging was carried out using a fluorescence microscope at specified excitation and emission wavelengths (IF488, EX 465–495 nm, EM 515–555 nm). Phosphorylation of histone H2AX at double-strand break (DSB) sites produces discrete nuclear γ-H2AX foci. For γ-H2AX foci quantification, 10 cells were randomly selected from each of five subculture-derived assay flasks nested within three founder lineages (50 cells per group in total; lineage distribution: one flask from lineage 1, two flasks from lineage 2, and two flasks from lineage 3). The mean number of foci per cell was first calculated within each assay flask, and assay-flask-level means were used for statistical interpretation. For PCNA fluorescence analysis, 50 cells were randomly selected from each of five subculture-derived assay flasks nested within three founder lineages (250 cells per group in total; lineage distribution: one flask from lineage 1, two flasks from lineage 2, and two flasks from lineage 3). The nuclear-to-cytoplasmic mean optical density (MOD) ratio was calculated for individual cells, then summarized at the assay-flask level for statistical analysis.

### 2.6. Phalloidin Staining

We fixed cells from the NP-30th, RCY-10th, and control groups with 4% paraformaldehyde for 20 min, followed by three washes with PBS. Membrane permeabilization was achieved by incubating the cells with 0.5% Triton X-100 at room temperature for 20 min, followed by three more PBS washes. We then stained the cells with IF488-conjugated phalloidin staining solution (Servicebio) for 120 min at room temperature in the dark. After three PBS washes, DAPI was applied for 10 min to stain the nuclei, followed by another three PBS washes. Finally, the samples were mounted with antifade mounting medium before imaging using the same fluorescence microscope described in Section 2.5.

We then captured images for DAPI (EX 330–380 nm, EM 420 nm), IF488 (EX 465–495 nm, EM 515–555 nm), and NPs (EX 535 nm; EM 610 nm). Five subculture-derived assay flasks nested within three founder lineages were assessed for each group (lineage distribution: one flask from lineage 1, two flasks from lineage 2, and two flasks from lineage 3), with 200 randomly selected cells analyzed per assay flask, totaling 1000 cells per group. Following the classification method of Verderame et al. [22], we categorized phalloidin-stained cells into four classes (Section 3.4) and statistically analyzed the percentage of cells in each class.

### 2.7. SEM and Transmission Electron Microscopy (TEM)

For SEM analysis of the NPs, 50 µL of the NP suspension was placed onto a slide with an anti-stripping coating and allowed to dry at room temperature. The specimens were then mounted onto metallic stubs using carbon adhesive tapes and sputter-coated with gold for 30 s using a Hitachi MC1000 Ion Sputtering Apparatus (Tokyo, Japan). Finally, the samples were observed, and images were captured using a Hitachi SU8100 SEM (Tokyo, Japan).

For SEM analysis of cells, we seeded cells from the NP-30th, RCY-10th, and control groups onto 20 mm round coverslips. Each group had three biological replicates. The LMR cells that adhered to the coverslips were fixed with 2.5% glutaraldehyde for 2 h. After three washes with 0.1 M phosphate buffer (PB), we post-fixed the cells with 1% osmium tetroxide in 0.1 M PB at room temperature for 2 h, followed by dehydration through a graded ethanol series. We then dried the samples and sputter-coated them with gold. The cell surface ultrastructure was observed using a Hitachi SU8100 SEM (Tokyo, Japan).

For TEM analysis, we collected cells from the NP-30th, RCY-10th, and control groups and fixed them with 2.5% glutaraldehyde for 30 min. After postfixation, dehydration, resin infiltration and embedding, polymerization, ultrathin sectioning, and staining, we examined the subcellular structures using a Hitachi HT7800 TEM. Representative local perinuclear-space widths were measured from selected TEM micrographs using ImageJ to provide a visual scale for the ultrastructural observations [18]. Because the number of TEM images with clearly traceable inner and outer nuclear membranes was limited, these measurements were used only as descriptive image annotations and were not used for statistical comparison.

### 2.8. 5-Ethynyl-2′-Deoxyuridine (EdU) Assay for Cell Proliferation

We seeded cells from the NP-30th, RCY-10th, and control groups onto 20 mm round coverslips and used the Click-iT EdU-488 Cell Proliferation Assay Kit (Servicebio) according to the manufacturer’s instructions to identify proliferating cells. This involved in vitro EdU labeling, fixation, permeabilization, and EdU click reaction to label newly synthesized DNA. We used fluorescence microscopy to capture images for DAPI, IF488, and NPs using the same excitation and emission wavelengths described in Section 2.5. Each group included five subculture-derived assay flasks nested within three founder lineages (lineage distribution: one flask from lineage 1, two flasks from lineage 2, and two flasks from lineage 3), and 10 random fields of view were analyzed per assay flask (50 fields of view per group) to calculate the percentage of EdU-positive (EdU^+^) cells.

### 2.9. Relative Gene Expression Assays

We collected LMR cells from the NP-30th, RCY-10th, and control groups, extracted total RNA using TRIzol reagent and synthesized complementary DNA using the PrimeScript™ RT Reagent Kit (Takara, Shiga, Japan). Specific primers for *H2AX*, mitogen-activated protein kinase 1 (*ERK1*), *PCNA*, and nucleoporin (NUP) genes (*NUP43*, *NUP50*, *NUP107*, *NUP133*, *NUP153*, *NUP160*, *NUP85*, *NUP53*, *NUP54*, *NUP93*, *NUP62*, *NUP88*, *NUP214*, and *NUP37*) were designed using Primer Premier 6 software (http://www.premierbiosoft.com/) (Table S1). We performed SYBR Green-based quantitative real-time polymerase chain reaction (qRT-PCR) on a Roche LightCycler 480 instrument (Basel, Switzerland) according to previously reported PCR protocols and reaction conditions [19] to measure the relative expression levels of H2AX, ERK1, and PCNA. We also performed RT-PCR to measure the relative expression levels of NUPs following the method described by D’Angelo et al. [23]. Glyceraldehyde-3-phosphate dehydrogenase (GAPDH) was used as the reference gene. Each experiment was conducted using five subculture-derived assay flasks nested within three founder lineages (lineage distribution: one flask from lineage 1, two flasks from lineage 2, and two flasks from lineage 3).

### 2.10. Biochemical Assays

We collected LMR cells from the NP-30th, RCY-10th, and control groups to measure the malondialdehyde (MDA) content, total superoxide dismutase (T-SOD) activity, and catalase (CAT) activity in the cells using assay kits obtained from Servicebio. All kit reactions and calculations were performed according to the manufacturers’ protocols. The protein concentration of the samples was measured using the bicinchoninic acid (BCA) protein quantification assay kit [24], and MDA content, T-SOD activity, and CAT activity were normalized to BCA-determined protein concentration. Each group included five subculture-derived assay flasks nested within three founder lineages (lineage distribution: one flask from lineage 1, two flasks from lineage 2, and two flasks from lineage 3).

### 2.11. Western Blot

LMR cells were collected from the NP-30th, RCY-10th, and control groups, and cytoplasmic proteins and total proteins were extracted using a cytoplasmic protein extraction kit (Beyotim, Shanghai, China) and lysis solution (Servicebio), respectively. The protein solution was mixed with 5 × loading buffer at a 4:1 ratio, denatured at 95 °C in a metal bath for 10 min, and subjected to sodium dodecyl sulfate–polyacrylamide gel electrophoresis and transfer onto a polyvinylidene fluoride membrane. The membranes were blocked with 5% non-fat milk at room temperature for 30 min, followed by overnight incubation at 4 °C with mouse anti-ERK1/2 polyclonal antibody, mouse anti-PCNA polyclonal antibody, and mouse anti-GAPDH polyclonal antibody (Servicebio). After washing the membranes with Tris-buffered saline containing Tween 20 (TBST), the membranes were incubated for 30 min at room temperature with horseradish peroxidase-tagged goat anti-rabbit IgG (heavy and light chains), followed by additional TBST washes. Finally, protein bands were visualized using enhanced chemiluminescence, and images were acquired after a 1 min exposure.

### 2.12. Statistical Analysis

Graphs were prepared using GraphPad Prism and Microsoft PowerPoint (version 10.6.1). Results are presented as mean ± standard deviation (SD). Before applying parametric tests, data distributions were inspected and preliminary assumptions were evaluated using the Shapiro–Wilk test for normality and Levene’s test for homogeneity of variance when at least three independent replicate-level values were available per group. No principal comparison required replacement by a non-parametric test after assumption checking; therefore, the reported inferential analyses were based on the parametric tests specified below, together with the founder-lineage-level sensitivity analyses for nested Experiment 2 endpoints. For two-group comparisons at a single passage or recovery time point, an unpaired two-tailed Student’s *t*-test was used when assumptions were met. For comparisons involving more than two groups, one-way analysis of variance (ANOVA) followed by Tukey’s multiple-comparison test was used. For multi-dose MTT assays, comparisons were conducted within each exposure duration. For image-based endpoints, individual cells or fields of view were treated as subsampling units; assay-flask-level means or percentages were calculated before statistical interpretation to avoid treating subsampling units as independent replicates. For Experiment 2 endpoints originally summarized from five assay flasks nested within three founder lineages, a founder-lineage-level sensitivity analysis was added. The five assay-flask values were aggregated into three founder-lineage-level values according to the pre-specified 1:2:2 structure: flask 1 = lineage 1, flasks 2–3 = lineage 2, and flasks 4–5 = lineage 3. For adjacent time-point comparisons, paired *t*-tests using founder lineage as a blocking factor were applied. For other nested n = 5 Experiment 2 endpoints, founder-lineage-level unpaired *t*-tests were used as a conservative sensitivity analysis. Replicate numbers, analysis units, and the specific statistical test applied to each principal comparison are described in the corresponding Methods sections, figure legends, and Table S2. Effect sizes, including Cohen’s d for two-group comparisons, eta-squared values for ANOVA-based comparisons, and raw mean differences for founder-lineage-level sensitivity analyses, were calculated for principal quantitative comparisons where appropriate group-level raw data were available and are summarized in Table S2. Statistical analyses were performed using SPSS (Version 19; IBM Corp., Armonk, NY, USA), GraphPad Prism, and custom spreadsheet/Python (version 3.10) calculations for the lineage-level sensitivity analyses. Statistical significance was set at *p* < 0.05.

## 3. Results

### 3.1. NP Characteristics and Distribution

**Figure 1:**
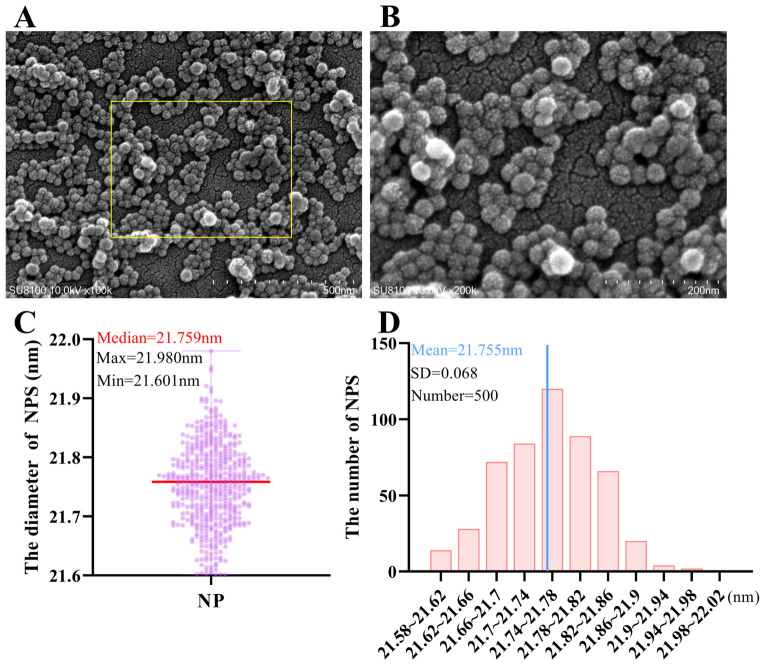
Morphology and size distribution of NPs. (**A**) Representative SEM image of NPs; (**B**) enlarged view of the yellow-boxed region in (**A**); (**C**,**D**) size distribution of 500 randomly measured NPs.

SEM imaging showed that the commercial NPs were predominantly spherical with smooth surfaces (Figure 1A,B). Based on measurements of 500 particles, NP diameters ranged from 21.601 to 21.980 nm, with a mean of 21.755 nm and a median of 21.759 nm, indicating a narrow size distribution consistent with the manufacturer’s specifications (Figure 1C,D). Minor aggregation observed in SEM images is likely attributable to particle drying during sample preparation.

DLS showed that the hydrodynamic diameter of NPs was approximately 49.72–54.79 nm in the aqueous dispersant and increased to approximately 88.10–95.16 nm in complete culture medium (Figure S2). This increase is consistent with adsorption of biomolecules and formation of a protein corona, together with ionic-strength-induced screening that can promote mild agglomeration in complex media.

To evaluate fluorescence stability, the PL spectra of the fluorescent microspheres were monitored under light-exclusion storage (35 days) and intermittent light exposure simulating cell passaging (35 days). No apparent shift in the maximum emission peak or spectral shape was observed, and the emission intensity remained largely stable, indicating that the fluorescence properties of the NPs were maintained during the experimental period (Figure S3).

### 3.2. NP Exposure Was Associated with Cytotoxicity and Prolonged Cell Doubling Time

**Figure 2:**
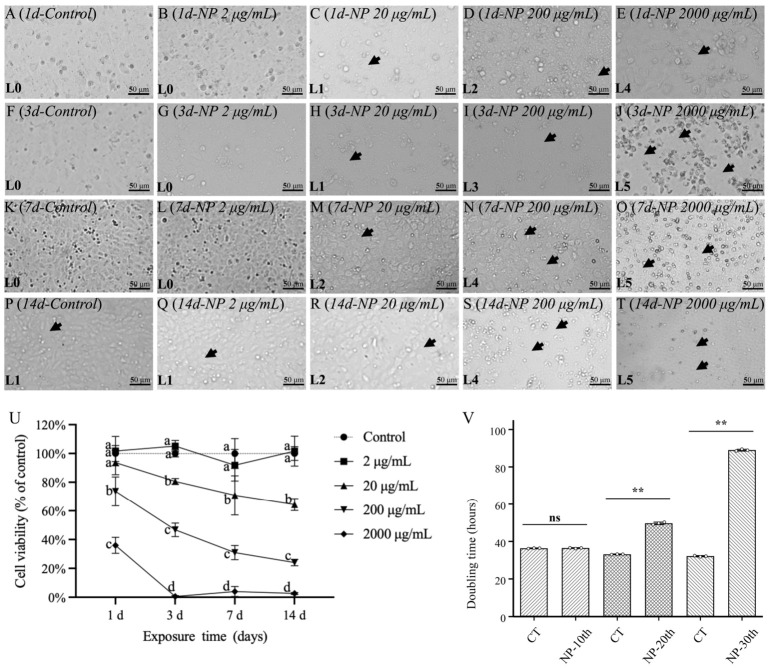
Cytopathic effects, cell viability, and proliferation changes in LMR cells after nanoplastic exposure. (**A**–**T**) Representative bright-field images of LMR cells exposed to NPs at 0 (Control), 2, 20, 200, and 2000 μg/mL for 1 day (**A**–**E**), 3 days (**F**–**J**), 7 days (**K**–**O**), and 14 days (**P**–**T**). Black arrows indicate representative cytopathic effects (CPEs), including cell rounding, shrinkage, and detachment. The cytotoxicity grade (L0–L5) is shown in the lower-left corner of each panel: L0, no cytotoxicity; L1–L5, increasing cytotoxicity based on reduced cell viability and the presence of CPEs, as defined in the Materials and Methods. Scale bars, 50 μm. (**U**) Relative cell viability of LMR cells after exposure to different NP concentrations for 1, 3, 7, and 14 days, determined by MTT assay. At each exposure time, the corresponding time-matched control was set to 100%, and all treatment values were normalized to that control. Data are presented as mean ± SD (n = 5 independent biological replicates; each replicate was seeded from a separately maintained T-25 culture flask in Experiment 1). Different lowercase letters indicate significant differences among concentrations at the same exposure time (*p* < 0.05). (**V**) Doubling time of LMR cells during long-term exposure to 20 μg/mL NPs at the 10th, 20th, and 30th passages. Nine assay-flask values per group were nested within three founder lineages (three assay flasks per lineage) and were averaged within lineage. Bars show the mean ± SD of the three founder-lineage means (n = 3), and open circles represent individual lineage means. Comparisons were performed using unpaired two-tailed Student’s *t*-tests on founder-lineage means. ns, not significant (*p* = 0.535); **, *p* < 0.01.

During short-term exposure, no obvious CPEs were observed in the control and 2 μg/mL NP groups within the first 7 days (Figure 2A,B,F,G,K,L). In contrast, CPEs appeared as early as day 1 in the 20, 200, and 2000 μg/mL NP groups, with severity increasing with concentration and exposure duration (Figure 2C–E). Specifically, on day 1 the cytotoxicity levels were graded as L1, L2, and L4 in the 20, 200, and 2000 μg/mL groups, respectively (Figure 2C–E). In the 20 μg/mL group, cytotoxicity remained at L1 during days 1–3 and increased to L2 at days 7–14 (Figure 2C,H,M,R). Mild CPE-like changes were also observed at day 14 in the control and 2 μg/mL groups (Figure 2P,Q). Consistently, cell viability showed a clear dose- and time-dependent decline at higher concentrations, with values normalized to the time-matched control within each exposure duration (Figure 2U).

Given the gradual cytotoxic progression observed at 20 μg/mL in Experiment 1, we selected this concentration for long-term exposure with serial passaging in Experiment 2. During continuous exposure to 20 μg/mL NPs, cell doubling time was assessed at the 10th, 20th, and 30th passages, corresponding to approximately 15, 55, and 105 days of exposure, respectively. At each passage, nine subculture-derived assay flasks per group were nested within three founder lineages (three assay flasks per lineage). To account for this structure, assay-flask values were averaged within each founder lineage, and inferential analyses used the three founder-lineage means. After 10 passages (NP-10th), the doubling time of NP-exposed cells did not differ significantly from that of the time-matched control (*p* = 0.535). After 20 passages (NP-20th), the doubling time was significantly prolonged (*p* < 0.001), and by 30 passages (NP-30th), it increased markedly to approximately 89 h, substantially longer than that of the corresponding control (*p* < 0.001; Figure 2V; Table S2). This delayed proliferative impairment may reflect cumulative cellular stress rather than a single acute cytotoxic event. In contrast, time-matched control cells showed a gradual decrease in doubling time across passages (Figure S4).

### 3.3. Accumulation and Apparent Loss of NP-Associated Fluorescence After Long-Term Exposure and Recovery Culture

**Figure 3:**
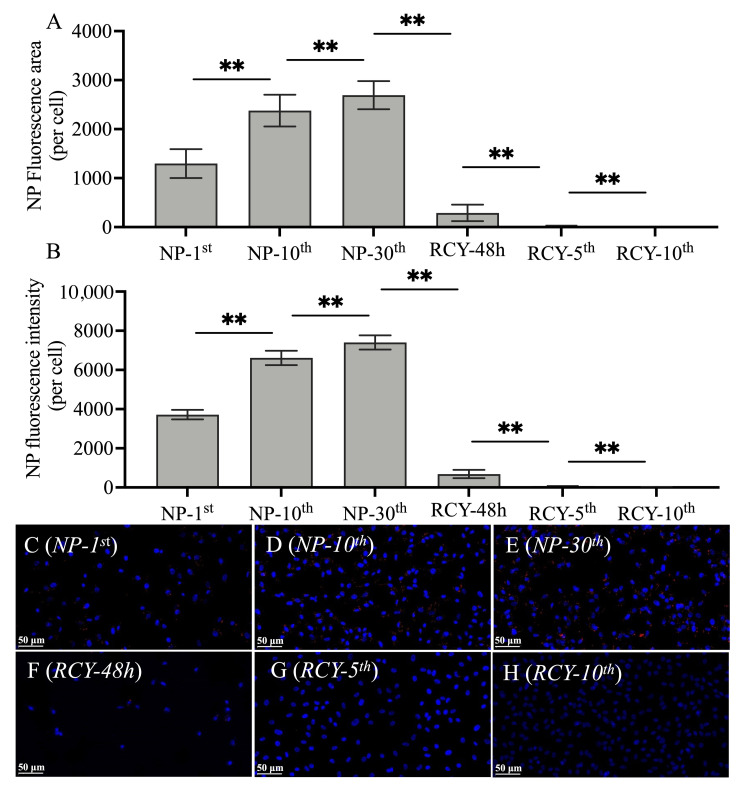
Accumulation and retention of NPs in LMR cells during prolonged exposure and after recovery. (**A**) NP fluorescence area per cell and (**B**) NP fluorescence intensity per cell at the 1st, 10th, and 30th passages during continuous NP exposure (NP-1st, 1.5 days; NP-10th, 15 days; NP-30th, 105 days) and after recovery following NP removal (RCY-48h; RCY-5th, 22 days; RCY-10th, 35 days). At each time point, 20 random fields were quantified for each of five assay flasks nested within three founder lineages (lineage distribution 1:2:2). Data are presented as mean ± SD of the five assay-flask-level means, and significance markers indicate assay-flask-level comparisons between adjacent time points (n = 5). Corresponding paired founder-lineage-level sensitivity analyses (n = 3) are reported in Table S2. **, *p* < 0.01. (**C**–**H**) Representative fluorescence images showing NP-associated fluorescence (red) and DAPI-stained nuclei (blue) in the NP-1st (**C**), NP-10th (**D**), NP-30th (**E**), RCY-48h (**F**), RCY-5th (**G**), and RCY-10th (**H**) groups. Scale bars, 50 μm.

In Experiment 2, we examined NP-associated fluorescence during long-term exposure to 20 μg/mL NPs across serial passaging and after cessation of NP addition (Figure 3 and Figure S5). Confocal microscopy showed that NP-derived fluorescence was widely distributed in the cytoplasmic region during exposure (Figure S5). In some cells, NP signals were enriched in the perinuclear region, forming a rim-like pattern around the nucleus, and occasional overlap with the DAPI-defined nuclear area was observed in merged images (Figure S5). However, fluorescence microscopy alone cannot distinguish particles located inside cells from particles attached to the cell surface or definitively confirm nuclear entry.

To quantify NP-associated fluorescence, we measured fluorescence area and fluorescence density per cell (Figure 3A,B). Both metrics increased from NP-1st to NP-10th and further to NP-30th, indicating progressive cell-associated fluorescence during continued exposure. After NP removal, the fluorescence signal markedly declined (Figure 3A,B). By RCY-10th, NP-associated fluorescence became indistinguishable from background under our imaging and analysis settings (Figure 3A,B,H). NP-associated fluorescence in NP-1st reached approximately half of that in NP-30th, indicating rapid early accumulation or cell association upon exposure (Figure 3A–C). In addition, after only 48 h in NP-free medium (RCY-48h), fluorescence area and intensity decreased by ~90% relative to NP-30th (Figure 3A,B,F). Together, these results demonstrate rapid accumulation of NP-associated fluorescence during early exposure and a pronounced loss of detectable fluorescence following recovery culture, which may reflect dilution through cell proliferation and/or cellular export processes.

### 3.4. Long-Term NP Exposure Altered Cell Morphology and Ultrastructure

**Figure 4:**
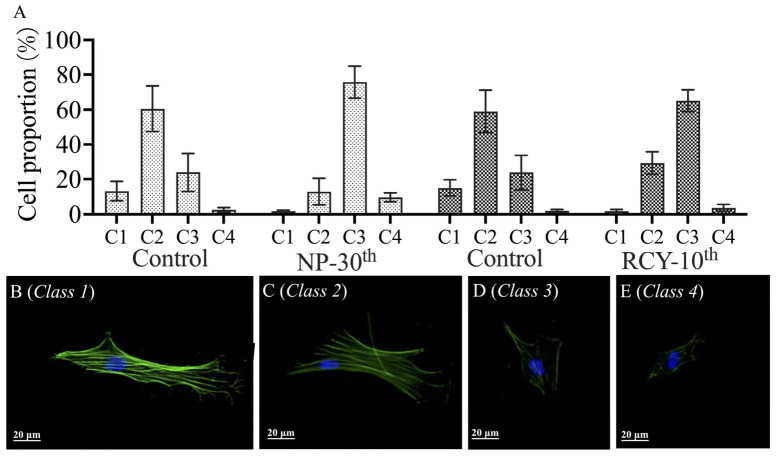
Reorganization of F-actin-defined cell morphology classes in LMR cells after long-term nanoplastic exposure and following recovery culture (RCY). (**A**) Proportions of F-actin-based morphological classes (Classes 1–4) in the time-matched control, NP-30th, and RCY-10th groups (classification criteria are described in Section 3.4). C1–C4 indicate Classes 1–4. Cells were randomly selected for classification (200 cells per assay flask; 1000 cells analyzed per group; five subculture-derived assay flasks nested within three founder lineages, lineage distribution 1:2:2). (**B**–**E**) Representative confocal images of Class 1–Class 4 cells showing F-actin organization (green) and nuclei (blue). Scale bars, 20 μm.

Following Verderame et al. [22], phalloidin-stained cells were categorized into four F-actin-defined morphological classes (Figure 4B–E). Class 1 cells contained prominent cables spanning more than 90% of the central cell area and covering at least 50% of the cell width. Class 2 cells contained fine cables and at least one prominent cable extending across more than half of the cell width into the central region. Class 3 cells contained only fine cables, whereas Class 4 cells lacked detectable cables in the central region and displayed diffuse fluorescence.

In the time-matched control groups for NP-30th and RCY-10th, Class 2 cells predominated, accounting for approximately 50–78% and 49–72% of cells, respectively (Figure 4A). In contrast, Class 3 cells predominated in the NP-30th group (62–84%), whereas Class 2 cells decreased to 6–23% (Figure 4A). After recovery (RCY-10th), Class 3 cells remained abundant (58–74%), whereas the proportion of Class 2 cells partially rebounded relative to NP-30th cells (22–39%) (Figure 4A), indicating incomplete restoration of F-actin organization.

SEM revealed that control cells displayed relatively smooth and intact surface structures. By comparison, NP-30th cells exhibited pronounced surface abnormalities, including cracks/fissures and ulcer-like lesions, accompanied by shortened and retracted filopodia; some filopodia also appeared fractured (Figure S6). In RCY-10th cells, surface morphology became more elongated and locally smoother, and regions with a more continuous membrane appearance were observed; however, residual localized damage remained in some areas (Figure S6).

**Figure 5:**
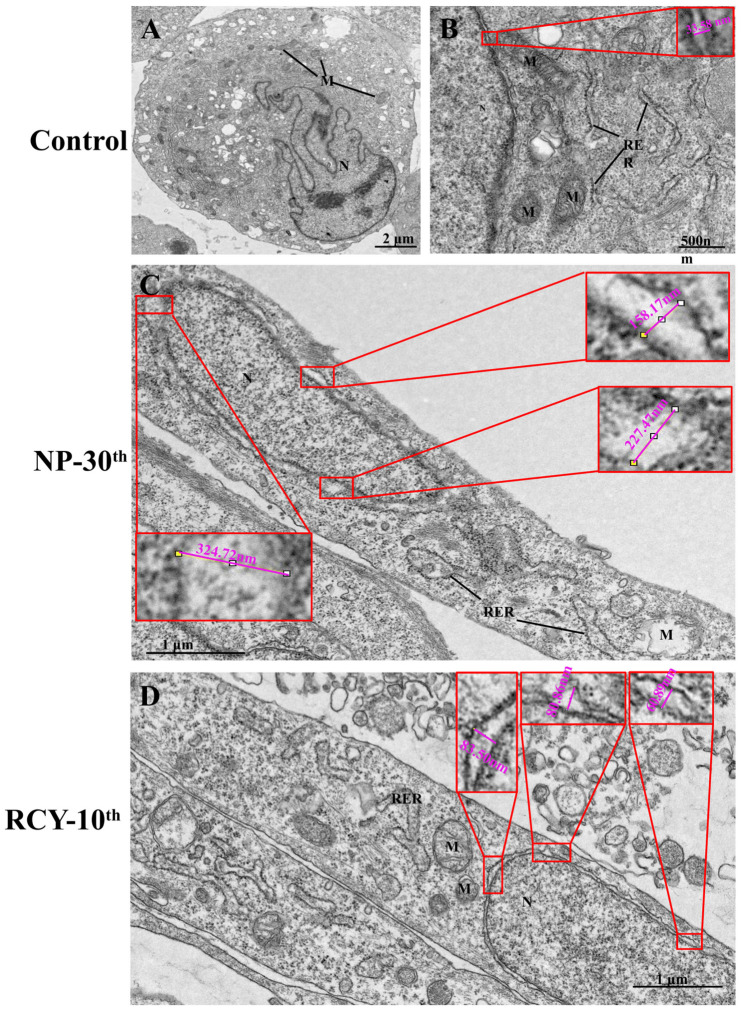
Representative TEM images showing subcellular ultrastructure in control (**A**,**B**), NP-30th (**C**), and RCY-10th (**D**) cells. Red boxes indicate magnified areas. Pink lines and values indicate representative local measurements of perinuclear-space width from the displayed TEM images; these values are provided for visual reference and were not used for statistical comparison. N, nucleus; M, mitochondria; RER, rough endoplasmic reticulum. Scale bars are as indicated.

Representative TEM images showed that control cells had continuous plasma membranes, abundant organelles, and an intact nuclear envelope with no obvious local widening of the perinuclear space; mitochondria were relatively uniform with preserved cristae and matrix (Figure 5A,B). In NP-30th cells, representative ultrastructural alterations included plasma membrane damage, organelle swelling, apparent local widening of the perinuclear space, mitochondrial membrane disruption with swollen morphology, and rough endoplasmic reticulum dilation with partial membrane dissolution and ribosome detachment (Figure 5C). In RCY-10th cells, some abnormalities were alleviated, but localized membrane damage, decreased cytoplasmic electron density, mild organelle swelling, apparent residual local widening of the perinuclear space, and mitochondrial membrane dissolution with reduced or disrupted cristae were still observed (Figure 5D). The local perinuclear-space values shown in Figure 5 are representative measurements from the displayed images and were not used for statistical comparison.

### 3.5. DNA Damage Signals Remained Elevated After a 10-Passage Recovery Period

We assessed DNA synthesis by EdU incorporation. The mean proportion of EdU-positive nuclei (EdU+ nuclei/total nuclei) was lower in NP-30th cells (30 passages, 105 days) than in the time-matched control, consistent with reduced DNA synthesis and slower proliferation. However, this endpoint did not remain statistically significant after founder-lineage-level sensitivity analysis (Table S2). After a 10-passage recovery period without NP exposure (RCY-10th), the EdU+ proportion was comparable to that of its time-matched control (Figure S7A–D; Table S2).

**Figure 6:**
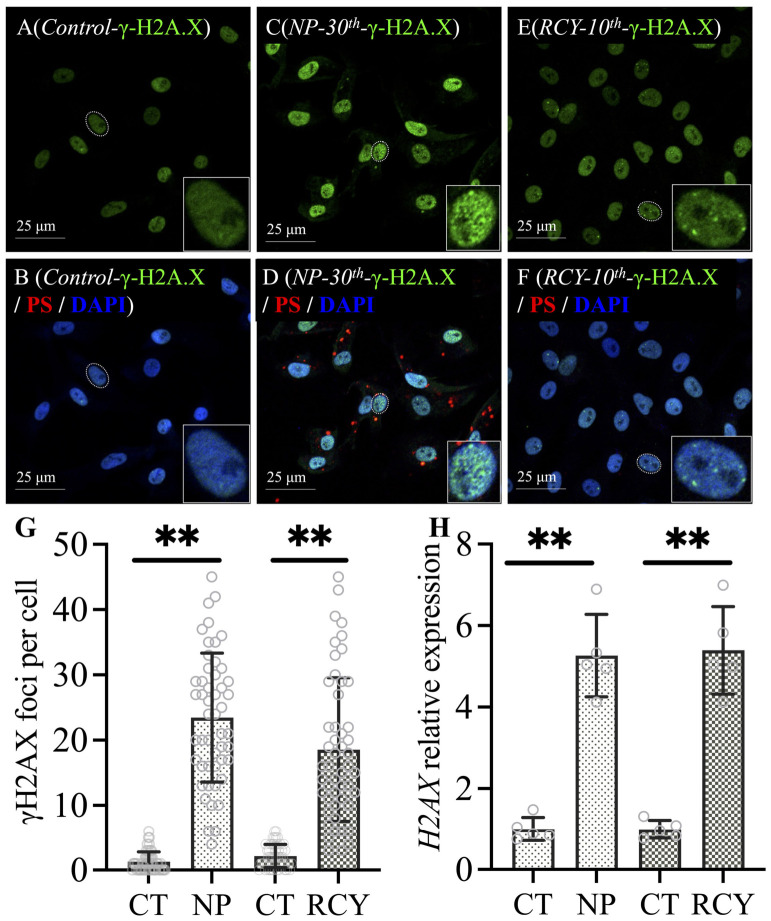
Residual nuclear damage signals in LMR cells revealed by γ-H2AX after long-term nanoplastic exposure and 10-passage recovery. (**A**–**F**) Representative immunofluorescence images of γ-H2AX foci in passage-matched control, NP-30th, and RCY-10th cells. Cells outlined by white dashed lines are magnified in the lower-right corner. Scale bar, 25 μm. (**G**) Quantification of γ-H2AX foci per cell. Ten cells were randomly selected from each assay flask, and individual-cell values are displayed to illustrate within-flask variation. (**H**) Relative transcriptional expression of H2AX. For both quantitative panels, five assay flasks nested within three founder lineages were analyzed per group (lineage distribution 1:2:2). Figure-level statistics and significance markers are based on the five assay-flask-level values (n = 5), whereas founder-lineage-level sensitivity analyses (n = 3) are reported in Table S2. Data are presented as mean ± SD. **, *p* < 0.01.

We further evaluated DNA double-strand break–related damage by immunofluorescence detection of γ-H2AX foci. Basal γ-H2AX foci were low in the two time-matched control groups, with mean values of 1.28 and 2.16 foci per cell, respectively (Figure 6G). In NP-30th cells, the mean number of γ-H2AX foci markedly increased to 23.46 per cell (Figure 6C,D,G). After recovery (RCY-10th), γ-H2AX foci decreased to 18.56 per cell but remained significantly higher than in the time-matched control group (Figure 6E–G). Consistently, *H2AX* transcript levels were significantly elevated in both NP-30th and RCY-10th groups compared with their respective controls (Figure 6H). Together, these results indicate residual DNA damage signals that remained detectable over the 10-passage recovery period after NP withdrawal.

Because oxidative stress is closely linked to DNA damage, we examined antioxidant-related endpoints. MDA content and CAT activity were significantly increased in NP-30th cells, whereas T-SOD activity remained unchanged. After the recovery period, MDA and T-SOD did not differ from their time-matched controls. CAT activity also showed no statistically significant difference from control after recovery (Figure S7E–G; Table S2).

### 3.6. Long-Term NP Exposure Altered NUP Transcription and Disrupted Nucleocytoplasmic Compartmentalization

**Figure 7:**
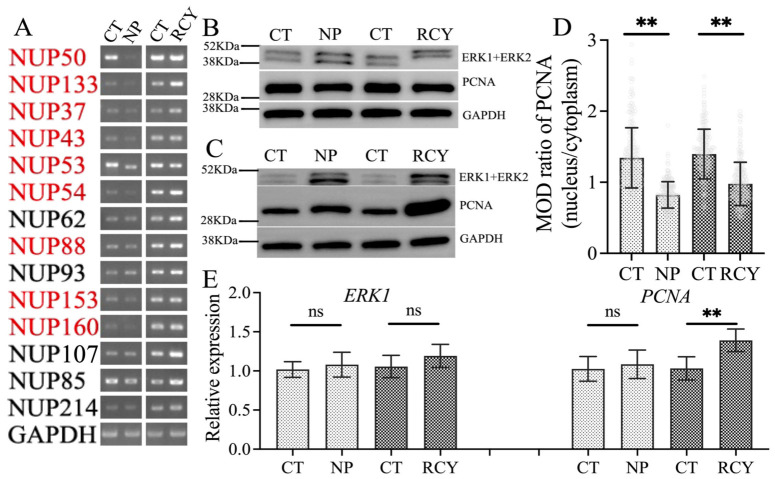
Nuclear health assessment in LMR cells at NP-30th and RCY-10th. (**A**) RT-PCR analysis of NUP gene expression in control (CT), NP-30th, and RCY-10th groups. Genes highlighted in red indicate lower expression in NP-30th than in the time-matched control. GAPDH was used as the reference gene. (**B**,**C**) Representative Western blots of ERK1/2 and PCNA in total protein extracts (**B**) and cytoplasmic fractions. (**C**) GAPDH was used as the loading control. These blots are presented as qualitative representative observations and were not used for statistical inference. (**D**) Nuclear-to-cytoplasmic mean optical density (MOD) ratio of PCNA. Fifty cells were randomly selected from each assay flask, and individual-cell values are displayed to illustrate within-flask variation. (**E**) Relative mRNA expression of ERK1 and PCNA determined by qRT-PCR. For panels (**D**,**E**), five assay flasks nested within three founder lineages were analyzed per group (lineage distribution 1:2:2). Figure-level statistics and significance markers are based on the five assay-flask-level values (n = 5), whereas founder-lineage-level sensitivity analyses (n = 3) are reported in Table S2. Data are presented as mean ± SD. ns, not significant; **, *p* < 0.01. CT, time-matched control; NP, NP-30th; RCY, RCY-10th.

To explore nuclear-associated alterations under long-term NP stress, we first examined the transcription of *NUPs*. After 30 passages of NP exposure (NP-30th), 9 of 14 *NUP* transcripts showed reduced expression relative to the time-matched control (Figure 7A). Following NP removal (RCY-10th), *NUP* transcription levels rebounded compared with NP-30th, with several transcripts approaching or exceeding control levels (Figure 7A), indicating partial transcriptional recovery.

We then assessed ERK1/2 and PCNA, which are nuclear-associated proteins capable of nucleocytoplasmic redistribution. In representative Western blots, total ERK1/2 and PCNA bands appeared broadly comparable among groups (Figure 7B), consistent with the qRT-PCR results showing no significant changes in ERK1 and PCNA transcripts in NP-30th cells compared with the time-matched control (Figure 7E). By contrast, cytoplasmic-fraction blots suggested stronger ERK1/2 and PCNA signals in NP-30th and RCY-10th cells than in their respective controls (Figure 7C). These representative protein patterns were interpreted together with the quantitative immunofluorescence-based analysis of PCNA distribution described below, which provided the principal quantitative evidence for altered nucleocytoplasmic distribution.

The quantitative evidence for altered PCNA distribution was obtained from immunofluorescence-based MOD analysis. The PCNA nuclear-to-cytoplasmic MOD ratio was significantly lower in NP-30th cells and remained lower in RCY-10th cells than in their respective controls, including in the founder-lineage-level sensitivity analysis (Figure 7D; representative images in Figure S8; Table S2), supporting residual disturbance in nucleocytoplasmic compartmentalization.

## 4. Discussion

### 4.1. Cellular Effects Emerge After Chronic Stress Exceeds a Threshold

Across an organism’s lifespan, cells can encounter plastic particles repeatedly, yet adverse outcomes may not be readily detectable in short- or medium-term assays. Although many studies have reported cytotoxic and genotoxic responses associated with nanoplastic exposure [19,25], fewer have examined how such responses evolve during continuous proliferation over extended passaging. In our study, the doubling time of LMR cells did not change significantly after 10 passages of exposure to 20 μg/mL particles (Figure 2V). However, by 20 passages, cell proliferation slowed markedly, and by passage 30, doubling time increased to nearly three times that of the time-matched control (Figure 2V). Similar threshold-like phenomena have been noted at the organismal level; for example, long-term nanoplastic/microplastic exposure in Daphnia magna can shorten lifespan and delay first reproduction, effects that may not be observed under acute exposure [26,27]. Together, these observations support the view that chronic exposure outcomes may become apparent only after a sufficient exposure duration or accumulated burden, underscoring the need for long-term assessments. Importantly, because bulk-polymer and chemical/extract (leachate) controls were not included, the present findings should be interpreted as responses associated with this specific 20 nm carboxylated fluorescent polystyrene particle formulation rather than definitive nanoscale-specific effects.

### 4.2. After Particle Fluorescence Became Undetectable, LMR Cells Showed Incomplete Recovery

Previous studies have shown that nanoscale particles may associate with cell membranes and enter cells through membrane penetration and/or endocytic internalization pathways, and that internalized polystyrene micro- and nanoplastics can also be released from cells after internalization [28,29]. During continuous exposure up to 30 passages, NP-associated fluorescence increased (Figure 3A–E), consistent with progressive cell association and/or uptake during repeated passaging. Notably, after particle removal, NP-associated fluorescence declined rapidly; within 48 h, both fluorescence-positive area and fluorescence intensity per cell decreased by approximately 90% (Figure 3A,B,F). Because 48 h is insufficient for most cells to complete a full division cycle, dilution by mitosis alone is unlikely to fully explain this rapid decline. In the present study, receptor involvement and signaling pathways were not directly tested; therefore, the fluorescence changes are interpreted as evidence of altered cell-associated particle burden rather than proof of a specific uptake/export mechanism. This pattern is consistent with the possibility that particle burdens may decrease via active export/exocytosis, in addition to dilution during cell division [29,30].

By the 10th recovery passage, NP-associated fluorescence was undetectable under the imaging conditions used (Figure 3A,B,H). EdU incorporation, MDA content, and T-SOD activity were comparable to their time-matched controls during recovery (Figure S7A–F). CAT activity remained numerically higher but was not statistically significant at either assay-flask or founder-lineage level; given the small number of founder lineages, this nonsignificant result should not be interpreted as evidence of equivalence (Figure S7G). However, several subcellular alterations remained detectable within the 10-passage recovery window despite the loss of detectable NP-associated fluorescence, including TEM-observed nuclear-envelope alterations, disorganization of perinuclear F-actin, and elevated DNA damage signaling (γ-H2AX foci) (Figure 4, Figure 5 and Figure 6).

The nuclear envelope consists of inner and outer membranes separated by a perinuclear space typically 30–50 nm [31,32], and its mechanical coupling to the cytoskeleton is mediated by actin-based structures and associated linker complexes [33]. Representative TEM images showed apparent nuclear-envelope alterations in NP-30th cells, including local widening of the perinuclear space, and residual alterations after 10 recovery passages (Figure 5). The local perinuclear-space values shown in Figure 5 are representative measurements from the displayed images and were not used for statistical comparison. Consistently, F-actin-defined morphology classes shifted strongly toward Class 3 under exposure and remained biased toward Class 3 during recovery (Figure 4A–E), indicating residual impairment in actin organization around the nucleus. These ultrastructural and proliferative changes may be secondary to combined membrane stress, oxidative stress, cytoskeletal remodeling, and impaired organelle homeostasis, although the causal pathway was not directly tested. Such nuclear envelope abnormalities can compromise nuclear architecture and are associated with disrupted gene regulation and cellular dysfunction [32,34].

A key cellular response to DSBs is phosphorylation of H2AX at damage sites, forming discrete γ-H2AX nuclear foci [35]. Here, γ-H2AX foci per cell were markedly increased in NP-30th cells and remained significantly elevated after 10 recovery passages (Figure 6A–G; Table S2), indicating residual DSB-associated nuclear stress responses after particle fluorescence became undetectable. Although previous studies have reported increased γ-H2AX foci in mammalian cells exposed to nanoplastics [36,37], evaluations of DSB-related signals during a defined recovery period after long-term exposure remain limited. Such recovery-phase assessment is important for estimating not only the magnitude but also the persistence of pollutant-associated cellular damage within the observation window.

F-actin has also been implicated in DSB repair processes, including chromatin mobility and repair-factor recruitment [38]. Therefore, residual actin disorganization after long-term exposure may plausibly constrain efficient DSB repair, a hypothesis that warrants further mechanistic testing.

### 4.3. Altered NUP Transcription and PCNA Redistribution Indicate Disturbed Nucleocytoplasmic Compartmentalization

The nuclear pore complex (NPC) is a large aqueous channel spanning the double membrane of the nuclear envelope and is assembled from >30 NUPs [23]. NPCs disassemble during mitosis and reassemble in daughter nuclei, while new pores can also form during interphase; both processes require appropriate NUP expression [23]. In our study, 9 of 14 tested *NUP* transcripts were reduced in NP-30th cells (Figure 7A), suggesting that prolonged exposure was associated with suppressed *NUP* transcription. After particle removal, *NUP* transcription rebounded in the RCY-10th group (Figure 7A). Insufficient *NUP* supply during extended proliferation could plausibly impair NPC assembly or maintenance, potentially weakening nucleocytoplasmic compartmentalization and increasing vulnerability to stress [39].

These analyses were performed at the NP-30th and RCY-10th time points, corresponding to 30 passages of NP exposure (105 days) and 10 particle-free recovery passages after exposure cessation, respectively. We further examined ERK1/2 and PCNA, which are nuclear-associated proteins involved in MAPK nuclear translocation, cell-cycle regulation, and DNA replication/repair [40,41]. Representative Western blots qualitatively suggested altered ERK1/2 and PCNA patterns in cytoplasmic fractions from NP-30th and RCY-10th cells (Figure 7B, C), but these blots were not quantitatively replicated and were therefore interpreted as supportive observations. The main quantitative support for altered nucleocytoplasmic distribution came from the immunofluorescence-based PCNA nuclear-to-cytoplasmic MOD ratio, which was reduced in both NP-30th and RCY-10th groups, including after founder-lineage-level sensitivity analysis (Figure 7D; Table S2), with representative PCNA immunofluorescence shown in Figure S8. Together, these results are consistent with residual alteration in nucleocytoplasmic compartmentalization under long-term exposure and incomplete recovery.

The significant increase in PCNA mRNA expression in the RCY-10th group may reflect a compensatory transcriptional response associated with recovery-phase DNA repair or renewed proliferative activity. However, because the PCNA nuclear-to-cytoplasmic MOD ratio remained reduced during recovery, increased PCNA transcription should not be interpreted as complete normalization of PCNA localization or nucleocytoplasmic compartmentalization.

### 4.4. Particle Characteristics and Study Limitations

Reported concentrations of micro- and nanoplastics vary widely among environmental and biological matrices and remain strongly dependent on the analytical method and particle-size range examined. For example, polystyrene particles smaller than 700 nm have been reported at concentrations of 47.75–1504.4 μg/L in the Pearl River basin [8], whereas micro- and nanoplastic burdens have also been detected in mussel tissues and human blood [42,43,44]. However, tissue or blood burdens are not directly equivalent to freely dispersed waterborne exposure concentrations. Differences in sample matrix, particle size, polymer composition, surface chemistry, analytical method, and reporting basis should therefore be considered when relating the nominal exposure concentration used in the present study to reported environmental concentrations and biological particle burdens. The 20 μg/mL concentration nevertheless provided an informative exposure condition for examining the cellular consequences of sustained NP exposure and subsequent recovery.

Cellular responses to plastic particles depend strongly on particle size, polymer chemistry, surface functionalization, and formulation components. Here, the tested material was a 20 nm carboxylated fluorescent polystyrene particle formulation; therefore, the observed nuclear and cytoskeletal alterations may reflect combined contributions from nanoscale size, carboxylated surface chemistry, embedded fluorescent dye, trace surfactant, residual monomers or oligomers, residual polymerization initiators, other additives, and extractables/leachates. Although the cultures were protected from light and the fluorescence-stability assay indicated stable particle fluorescence under the tested light-exclusion and intermittent-exposure conditions, we did not directly evaluate whether the embedded red dye could generate reactive oxygen species during repeated incidental light exposure. We also did not test the trace surfactant or particle-free leachates/extracts at the concentrations present after dilution to 20 μg/mL. In addition, the DLS data presented here are intensity-weighted distributions, which can overemphasize larger aggregates relative to their numerical abundance; number- or volume-weighted distributions were not available from the archived dataset. Finally, among the endpoints subjected to founder-lineage-level sensitivity analysis, the CT versus NP-30th comparison for EdU-positive cells was the only result for which statistical significance was not retained after the five assay-flask values were aggregated into three founder-lineage means. Although the direction of the reduction remained unchanged, this EdU result should be interpreted as supportive rather than definitive evidence of reduced proliferation and considered together with the doubling-time data. Future studies incorporating bulk-polymer particles, non-fluorescent particles, dye-only controls, surfactant controls, particle-free leachate/extract controls, and monomer/oligomer measurements will be necessary to separate size-specific effects from formulation chemistry and to strengthen environmental relevance.

## 5. Conclusions

Long-term exposure of LMR cells from spotted sea bass (*L. maculatus*) to nominal 20 nm carboxylated fluorescent polystyrene particles was associated with slowed proliferation, altered cell-surface and subcellular ultrastructure, and pronounced nuclear stress markers. After NP addition was stopped and cells were passaged for 10 additional particle-free passages, NP-associated fluorescence became undetectable and several endpoints partially recovered. However, TEM-observed nuclear-envelope alterations, F-actin reorganization, and elevated γ-H2AX foci remained detectable throughout the 10-passage recovery period, indicating incomplete recovery within the timeframe examined. These findings underscore the value of long-term exposure–recovery designs for evaluating plastic-particle hazards. Because bulk-polymer and particle-free chemical extract or leachate controls were not included, the observed effects cannot be attributed solely to nanoscale size. Future studies should combine chemical characterization of formulation-related compounds in stock suspensions and culture media with matched material, leachate, and chemical controls to distinguish particle-size effects from formulation-related effects.

## Data Availability

Data are contained within the article and Supplementary Materials.

## Supplementary Materials

The following supporting information can be downloaded at: https://www.mdpi.com/article/10.3390/toxics14070628/s1, Table S1. Primer sequences used in this study. Table S2. Summary of statistical tests, effect sizes, and founder-lineage-level sensitivity analyses for principal quantitative comparisons. Figure S1. Experimental design for short-term NP screening, long-term serial passaging, and recovery assessment in LMR cells. The study comprised three phases. (1) In the short-term screening experiment, LMR cells were seeded in 96-well plates and exposed to 0, 2, 20, 200, or 2000 μg/mL NPs for 1, 3, 7, or 14 days. Based on the gradual cytotoxic response observed during screening, 20 μg/mL was selected for prolonged exposure. (2) For long-term serial passaging, the time-matched control and NP-exposed groups were each initiated from three independent founder lineages. Cells were counted and reseeded at a fixed density at each passage, and samples were collected at NP-1st (1.5 days), NP-10th (15 days), and NP-30th (105 days). (3) After the 30th NP-exposure passage, NP addition was discontinued and cells were serially passaged for 10 additional passages in particle-free medium. Recovery samples were collected at RCY-48 h, RCY-5th (22 days), and RCY-10th (35 days). Time-matched controls were maintained in parallel throughout the long-term exposure and recovery periods. The principal endpoints included cell viability and morphology, NP-associated fluorescence, cellular ultrastructure, proliferation and DNA-damage markers, and nuclear molecular responses. Figure S2. DLS-measured hydrodynamic diameter of NPs. Intensity-weighted size distributions of 20 nm COOH-PS NPs (20 μg/mL) dispersed in water (H_2_O) or complete culture medium are shown (n = 3 independent measurements per condition). Z-average diameters were 47.69–47.82 nm in H_2_O and 73.51–73.66 nm in complete medium; the main intensity peak (Peak 1) ranged from 49.72–54.79 nm (H_2_O) and 88.10–95.16 nm (complete medium). Because intensity-weighted DLS distributions can overemphasize larger particles or aggregates, these data should be interpreted primarily as evidence of hydrodynamic size and aggregation tendency under the tested dispersion conditions. Figure S3. PL spectra of NPs. The PL spectra of the culture medium (black line), initial NPs (green line), and NPs after 35 days under full light exclusion (blue line) and 35 days of intermittent light exposure (simulating 10 rounds of cell passaging, 3 min per exposure, total 30 min) are shown in Figure S4. Figure S4. Doubling time of control cells at different time points. Data are presented as mean ± SD (n = 9). Different letters indicate statistical significance (*p* < 0.05). Figure S5. Representative high-magnification images illustrating NP-associated fluorescence patterns in LMR cells after long-term exposure. Yellow arrows indicate NP signals in the perinuclear region. White arrows indicate NP signals overlapping with the nuclear (DAPI) signal in the merged image (apparent co-localization). Fluorescence microscopy alone cannot distinguish intracellular signals from cell-surface-associated particles or confirm intranuclear localization. Scale bars, 20 μm. Figure S6. Surface ultrastructure of LMR cells revealed by SEM after prolonged nanoplastic exposure and recovery culture. (A) Representative SEM image of control cells showing intact cell surface morphology. (B–D) Representative SEM images of NP-30th cells showing surface damage and altered filopodia morphology; boxed regions indicate areas shown at higher magnification. (E–F) Representative SEM images of RCY-10th cells; boxed regions indicate magnified areas highlighting surface features after recovery culture. Arrow/marker definitions: yellow arrows indicate cell surface cracks; red arrows indicate cell surface ulcers; black arrows indicate retraction of filopodia; green dashed circles indicate filopodia breakage; white dashed circles indicate repaired cell membrane. Scale bars, 20 μm. Figure S7. Genotoxicity and oxidative-stress endpoints in LMR cells at NP-30th and RCY-10th. (A–C) Representative fluorescence images showing EdU incorporation (green), NP-associated fluorescence (red), and DAPI-stained nuclei (blue) in the control, NP-30th, and RCY-10th groups. Scale bar, 50 μm. (D) Percentage of EdU-positive nuclei. Ten randomly selected fields were analyzed in each of five assay flasks nested within three founder lineages (50 fields per group; lineage distribution 1:2:2), and data are presented as mean ± SD of the five assay-flask-level percentages. (E–G) MDA content (E), total SOD activity (F), and CAT activity (G). Data are presented as mean ± SD of five assay flasks nested within three founder lineages (lineage distribution 1:2:2). Significance markers in (D–G) represent assay-flask-level comparisons (n = 5); founder-lineage-level sensitivity analyses (n = 3) are reported in Table S2. The CT versus NP-30th difference in EdU-positive nuclei did not remain statistically significant in the founder-lineage-level analysis and is therefore interpreted as supportive evidence. ns, not significant; **, *p* < 0.01. Figure S8. PCNA immunofluorescence in LMR cells following prolonged nanoplastic exposure and recovery. Representative fluorescence images of PCNA staining in control (CT), NP-30th, and RCY-10th groups. (A–C) CT group: DAPI (A), PCNA (B), and merged image (C). (D–F) NP-30th group: DAPI (D), PCNA (E), and merged image (F). (G–I) RCY-10th group: DAPI (G), PCNA (H), and merged image (I). Cells outlined by white rectangular boxes are magnified and shown in the lower-right corner of each panel. Nuclei were counterstained with DAPI (blue), and PCNA is shown in green. Scale bar, 50 μm.
