# Supplementary figures and images for: Long-Term Exposure–Recovery to 20 nm Polystyrene Nanoplastic Particles Is Associated with Residual Nuclear Stress in a Marine Fish Cell Line
Source: Toxics. 2026 Jul 20;14(7):628. doi: 10.3390/toxics14070628 (PMC13431394; doi:10.3390/toxics14070628)

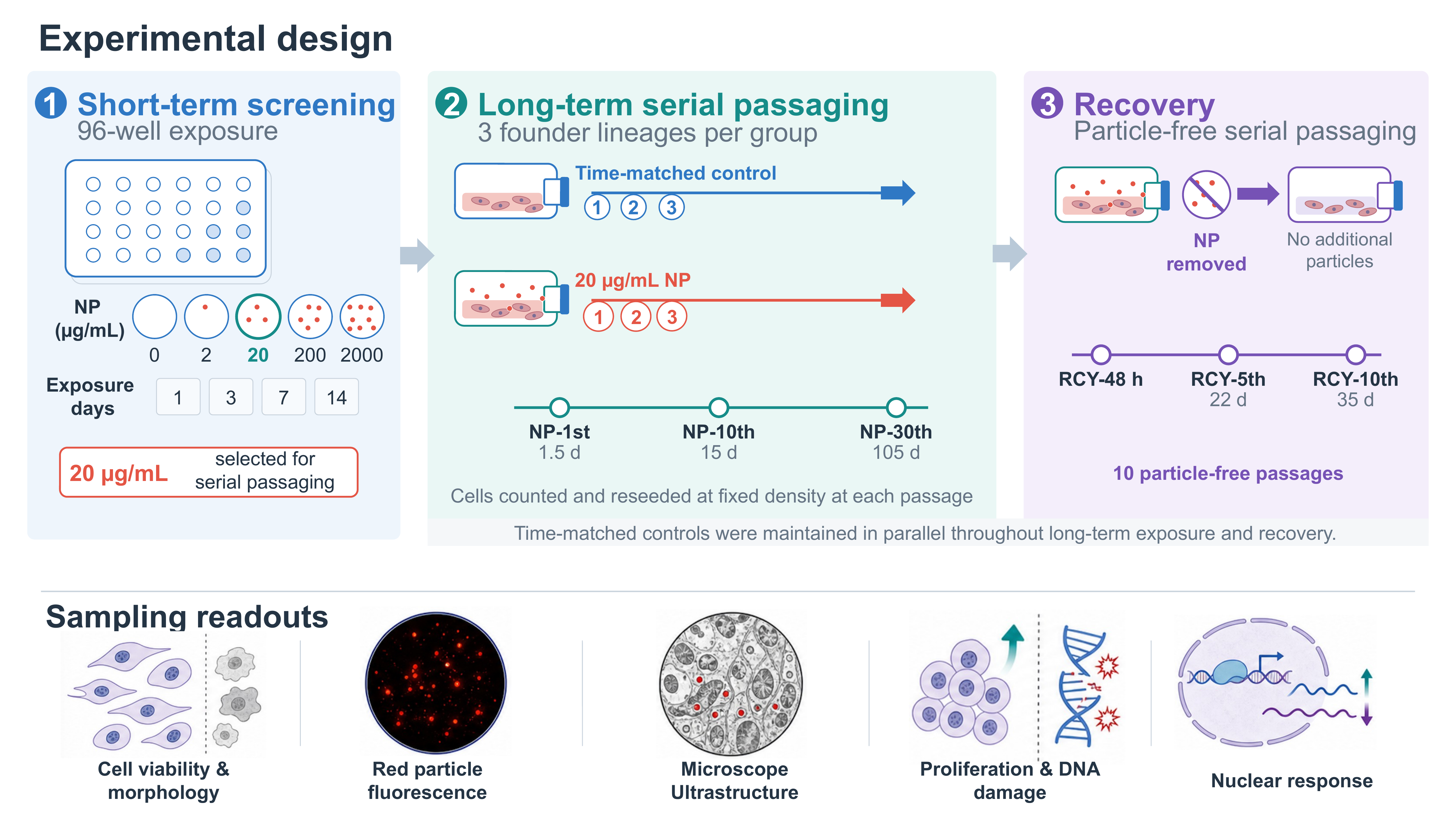

Supplement: Supplementary file 1 [file toxics-14-00628-s001.zip › Figure S1_experimental_design.tiff]

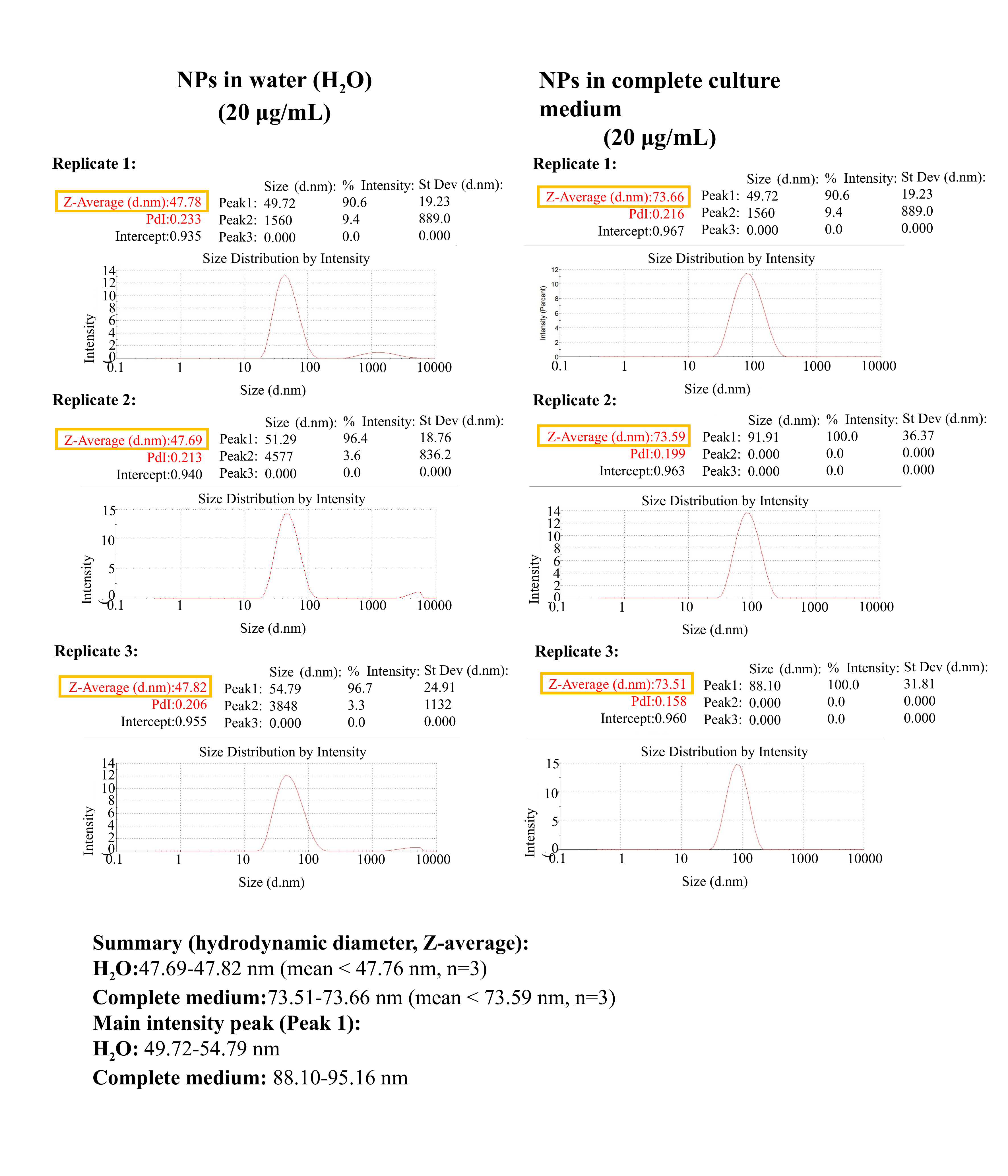

Supplement: Supplementary file 1 [file toxics-14-00628-s001.zip › Figure S2_DLS.tiff]

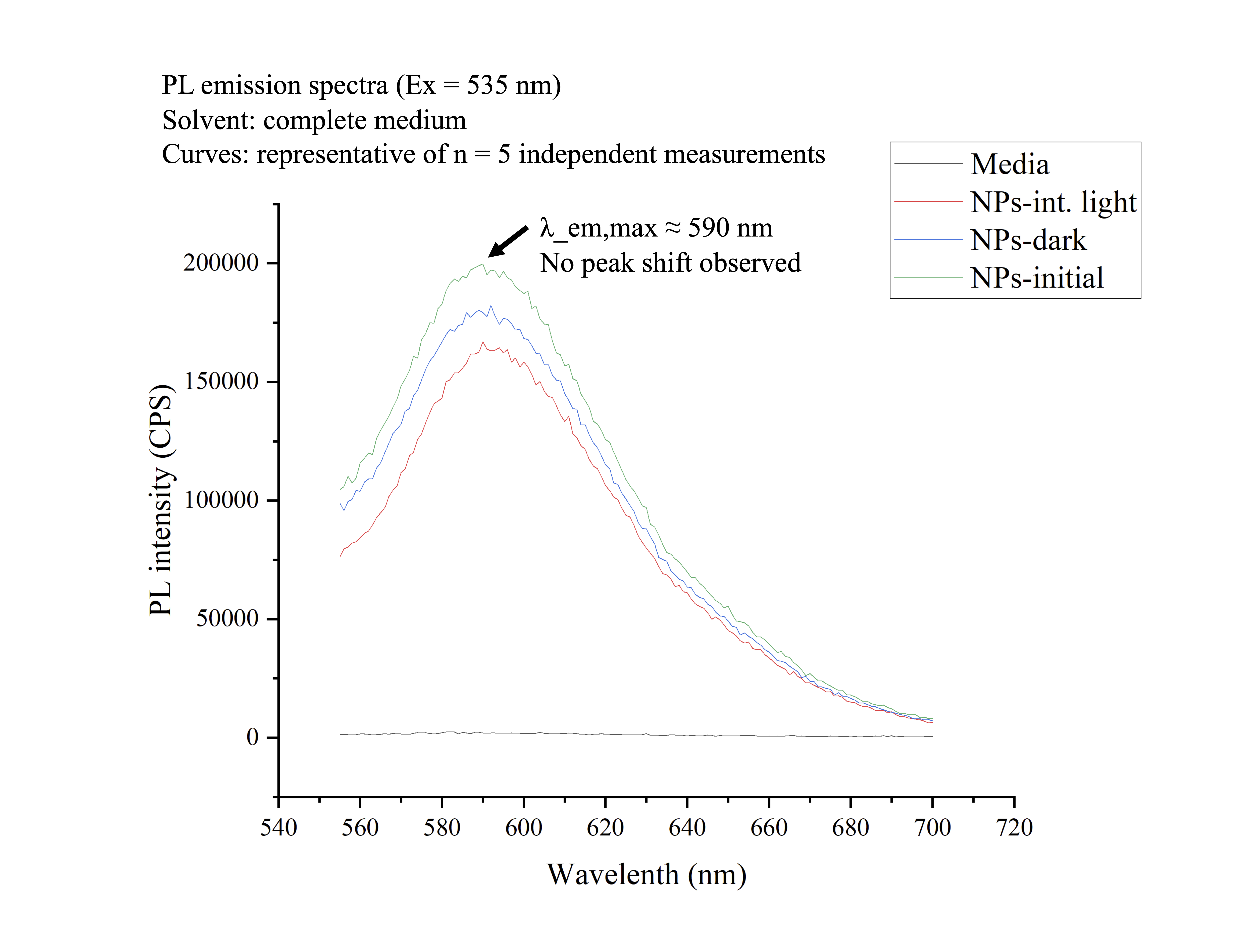

Supplement: Supplementary file 1 [file toxics-14-00628-s001.zip › Figure S3_PL.tiff]

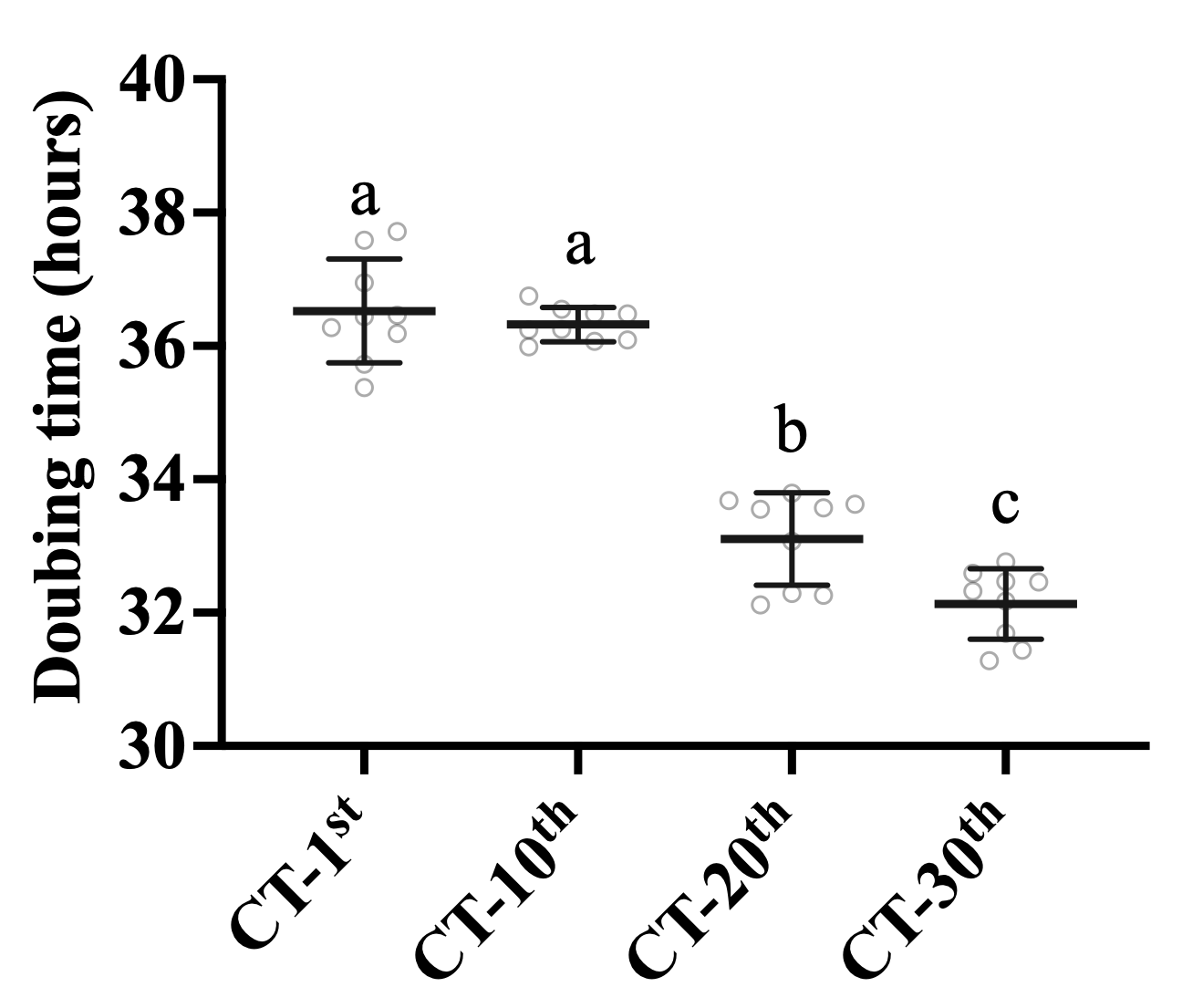

Supplement: Supplementary file 1 [file toxics-14-00628-s001.zip › Figure S4_control cells.tiff]

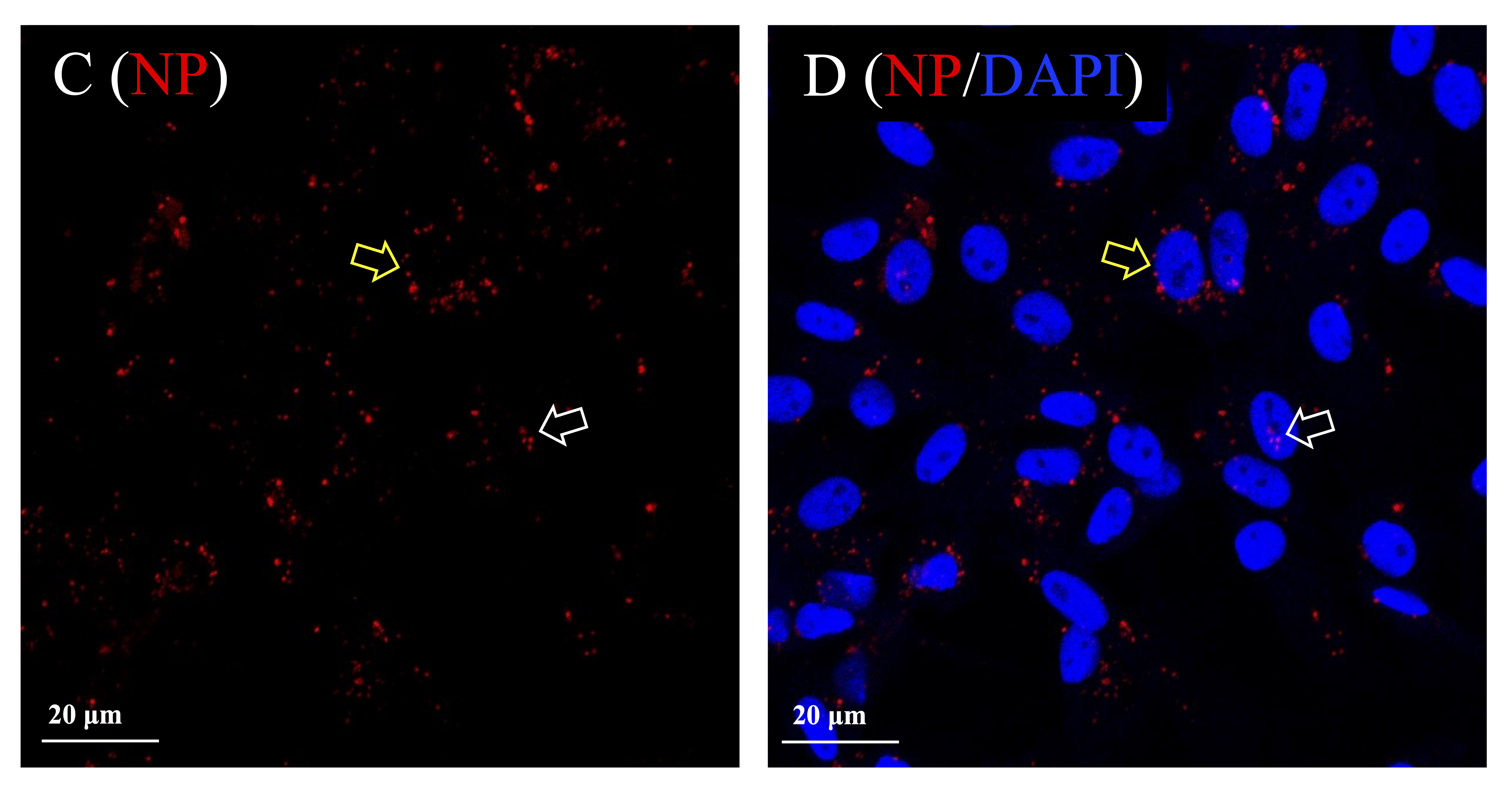

Supplement: Supplementary file 1 [file toxics-14-00628-s001.zip › Figure S5_np signal.tiff]

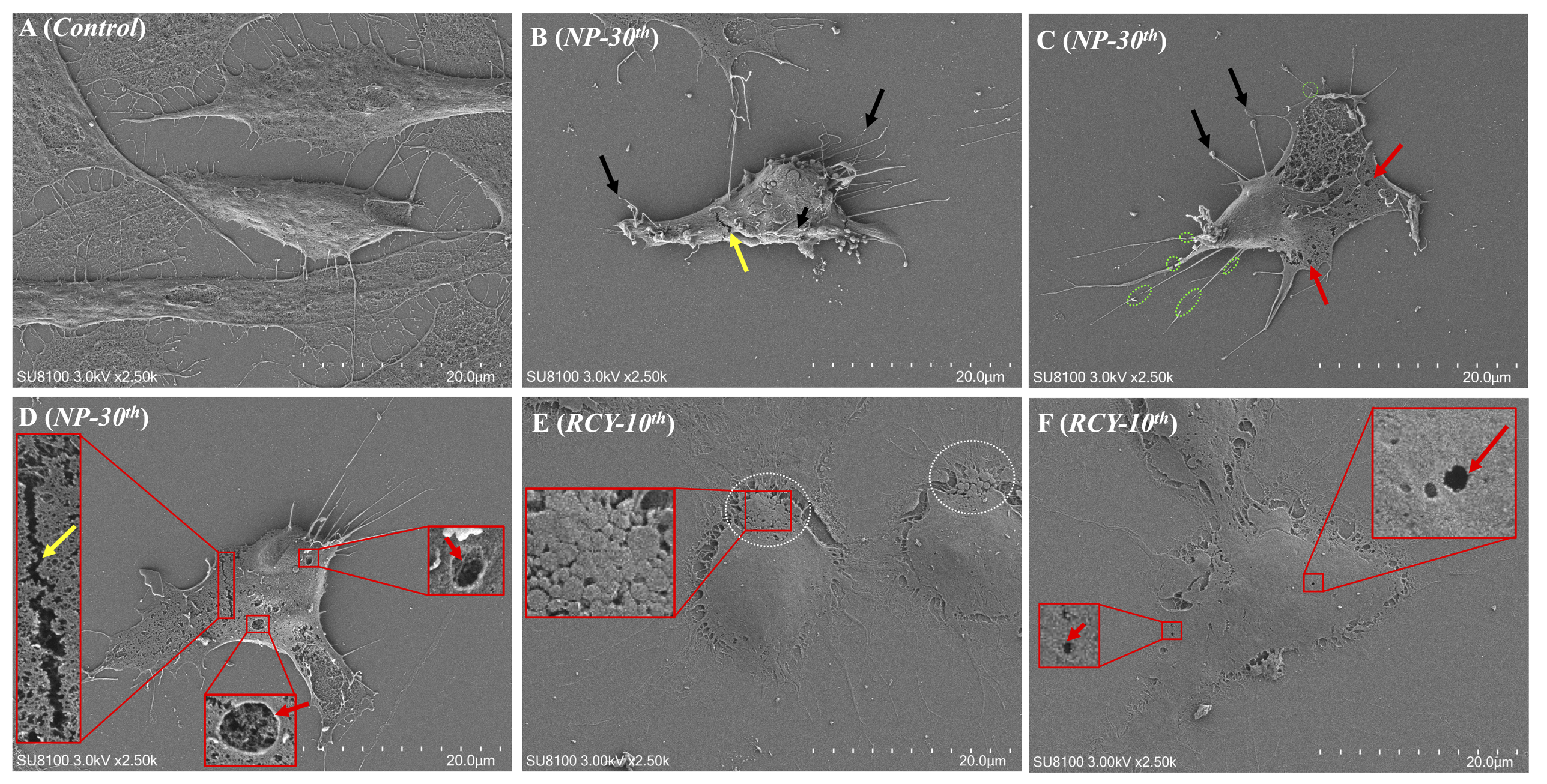

Supplement: Supplementary file 1 [file toxics-14-00628-s001.zip › Figure S6_SEM.tiff]

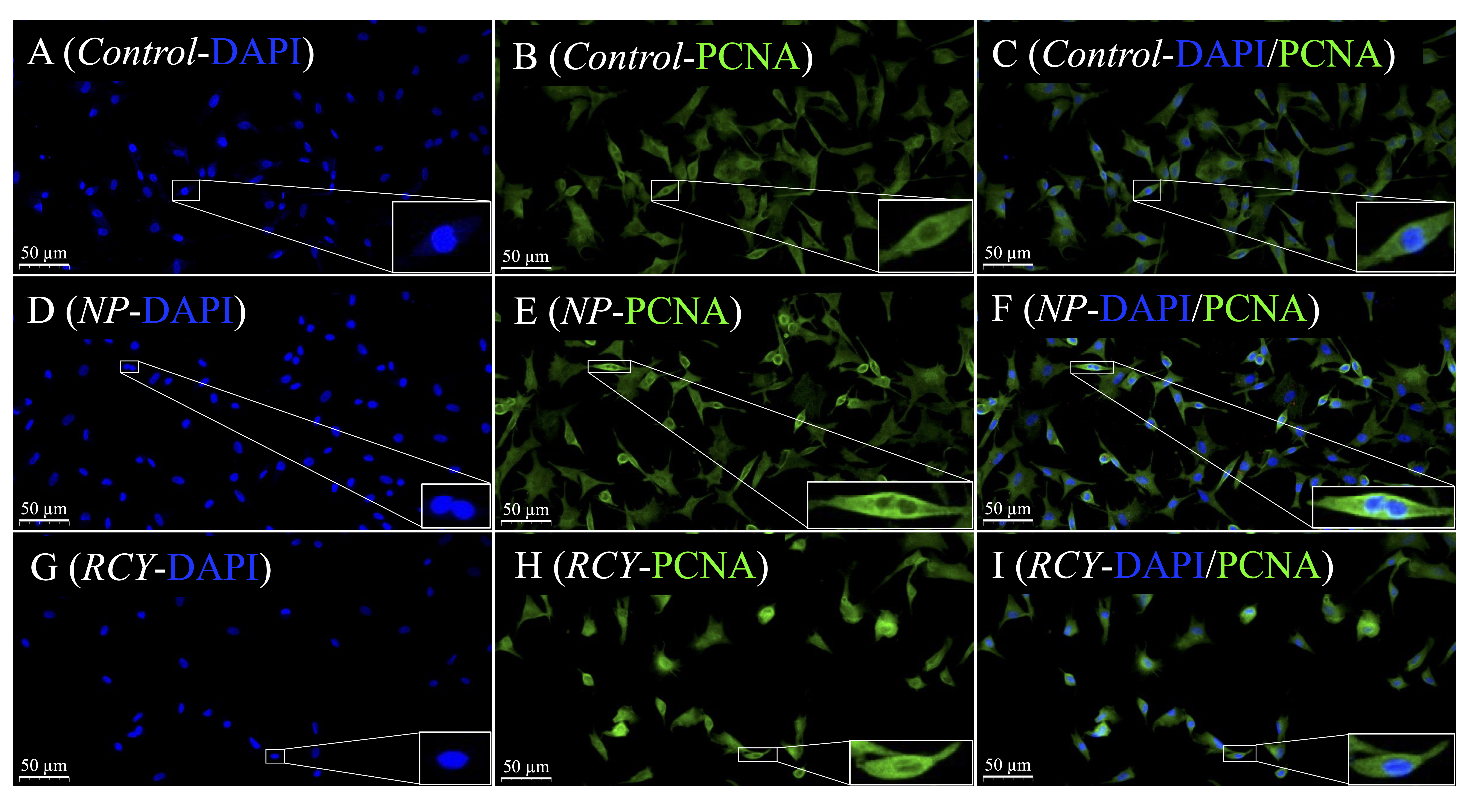

Supplement: Supplementary file 1 [file toxics-14-00628-s001.zip › Figure S8.tiff]
